# Naegele Forceps Delivery and Association between Morbidity and the Number of Forceps Traction Applications: A Retrospective Study

**DOI:** 10.1155/2015/483195

**Published:** 2015-09-03

**Authors:** Naoki Matsumoto, Toshifumi Takenaka, Nobuyuki Ikeda, Satoshi Yazaki, Yuichi Sato

**Affiliations:** Department of Obstetrics and Gynecology, Tatedebari Sato Hospital, 96 Wakamatsucho, Takasaki, Gunma 370-0836, Japan

## Abstract

*Objective*. To present the method of Naegele forceps delivery clinically practiced by the lead author, its success rate, and morbidity and to evaluate the relationship between morbidity and the number of forceps traction applications. *Methods*. Naegele forceps delivery was performed when the fetal head reached station +2 cm, the forceps were applied in the maternal pelvic application, and traction was slowly and gently performed. In the past two years, Naegele forceps delivery was attempted by the lead author in 87 cases, which were retrospectively reviewed. *Results*. The numbers of traction applications were one in 64.7% of cases, two in 24.7%, and three or more in 10.7%. The success rate was 100%. No severe morbidity was observed in mothers or neonates. Neonatal facial injury occurred most commonly in cases with fetal head malrotation, elevated numbers of traction applications, and maternal complications. Umbilical artery acidemia most commonly occurred in cases with nonreassuring fetal status. The significant crude odds ratio for three or more traction applications was 20 in cases with malrotation. *Conclusion*. Naegele forceps delivery has a high success rate, but multiple traction applications will sometimes be required, particularly in cases with malrotation. Malrotation and elevated numbers of traction applications may lead to neonatal head damage.

## 1. Introduction

Recently, the very high rate of cesarean delivery has been a topic of discussion and is considered a problem that should be solved [1]. In 2011, the cesarean delivery rate was 33% of all births in the United States [2]. With the increasing rate of cesarean delivery, the rate of operative vaginal deliveries has decreased during the past 20 years [2]. In Japan, the overall rate of cesarean delivery in 2011 was 19% [3] and that at high-level medical facilities was 34% [4]. In operative vaginal delivery, the rate of forceps delivery has decreased more than that of vacuum extraction [5]. In Japanese high-level medical facilities, the rates of cesarean, vacuum, and forceps delivery are 20%, 6%, and 1%, respectively, among all deliveries except for planned cesarean deliveries [4].

Thus, forceps delivery has become a minor obstetrical method in management of labor and delivery. However, the lead author considers that forceps delivery is his first-choice method in the operative vaginal deliveries. Forceps delivery has a higher success rate than vacuum extraction [6] and affords robust reliability for an experienced operator. Nonetheless, the author always bears in mind the potential risks of forceps. Therefore, he applies the forceps sufficiently in a gentle and slow manner to avoid undue maternal and neonatal morbidity. Sometimes multiple traction applications are needed. Some obstetricians believe that forceps delivery should be completed by one forceps' traction application [7]. However, no recent study has assessed the correlation between morbidity and the number of forceps traction applications.

The aim of this study was to present the method of Naegele forceps delivery clinically practiced by the lead author and its success rate and morbidity and moreover to evaluate the relationship between morbidity and other factors including the number of forceps traction applications.

## 2. Methods

### 2.1. Delivery Procedures with Naegele Forceps

Naegele forceps are most commonly used for forceps delivery in Japan. They have adequate pelvic and cephalic curves for nonrotational forceps delivery and fenestrated blades that permit firmer grasp of the fetal head [5]. Furthermore, Japanese obstetricians commonly use Naegele forceps modified and ameliorated for the Japanese women, which are called UTokyo Naegele forceps. They have lighter weight (417 g), shorter length (35 cm), and thinner blades than the original. Indications, prerequisites, and precautions for forceps delivery that were stipulated in the guidelines for obstetrical practice in Japan [8] were followed. In principle, forceps delivery was performed when the leading point of the fetal head reached or nearly reached station +2 cm over the ischial spine. The measurement of the station was performed based on internal digital examination in the dorsal position. The station was determined as the distance on the pelvic axis from the ischial spine to the leading point of the fetal head. Before application of forceps delivery, fetal head rotation and spine position were checked by internal digital examination as well as abdominal (sometimes with transperineal) ultrasonography. Based on this assessment, fetal head malrotation was diagnosed and classified as the occiput transverse position (with rotation greater than 45 degrees) or occiput posterior position. On the decision of forceps delivery, verbal informed consent was obtained. The option of primary cesarean delivery without trial of operative vaginal delivery was presented to the patient especially when the forceps trial was considered to have a possibility of failure and relatively high risk of maternal and neonatal morbidity. Rotation with Kielland rotational forceps was attempted when it was considered that it might effectively improve malrotation. In this study, it was attempted in three cases. Effective improvement of malrotation was obtained in one case, which was excluded from this study, but not in the other two cases, which were followed by Naegele forceps delivery and were included in this study. Naegele forceps were applied in the maternal pelvic application. After articulation of the forceps, the operator (the lead author) suspended the hooks on the first and middle fingers and placed the handles on the palm of his right hand with an underhand grip. He did not usually hold the handles. To feel the exact progression and to avoid sudden emergence, the tips of the fingers of the left hand were placed on the fetal head. To avoid falling, he adopted a fighter's stance with a wide stance and slightly bent knees. Before genuine traction, test traction was applied to check the forceps grip, fetal head movement, and feeling of fetal descent. Except in urgent situations, forceps traction was applied slowly and gently in synchrony with contractions and pushing efforts to avoid undue maternal and neonatal damage and forceps slipoff. Traction was directed, as per principle, along the axis of the birth canal with no rotational movement. Episiotomy (midline or mediolateral) was performed if necessary. The forceps were disarticulated when the operator considered the fetal head would not recede. The operator dictated a midwife to support the delivery by perineal protection when required. Forceps application was halted when the operator felt no evidence of progressive descent with three traction applications at most.

### 2.2. Study Design

**Figure 1:**
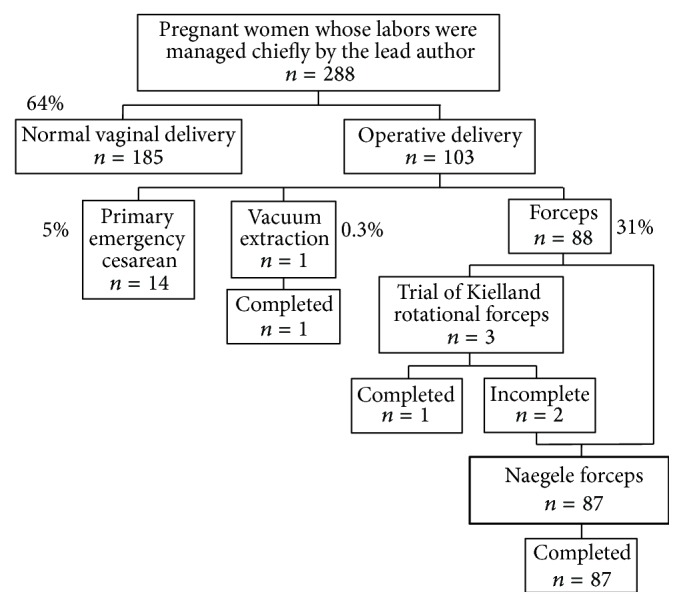
Mode of delivery of 288 term pregnant women with live singleton pregnancies and cephalic presentation whose labor and delivery were managed chiefly by the lead author.

The study period was the past two years from July 2012 to June 2014. In this period, 288 term pregnant women with live singleton pregnancies and cephalic presentation were managed chiefly by the lead author. In 87 of these cases, Naegele forceps delivery was attempted and successfully completed (Figure 1). We retrospectively reviewed the 87 cases and obtained patient characteristic factors, parturition outcomes, and short-term (during a period of one month after delivery) maternal and neonatal morbidity outcomes from the medical records.

The relationships between characteristic factors and morbidity were assessed by Fisher's exact test. The five morbidity outcomes considered were as follows: maternal anal sphincter injuries, acute postpartum urinary retention lasting over 24 h, dehiscence or maternal injuries except for perineal lacerations, neonatal facial injuries, and umbilical artery acidemia. Anal sphincter injuries were defined as third- or fourth-degree perineal lacerations. Acute postpartum urinary retention was defined as a postvoid residual volume of >100 mL. Neonatal facial injuries were defined as forceps marks with bruising or skin lacerations. Umbilical artery acidemia was defined as acidemia with umbilical artery pH <7.2. The relationship between characteristic factors and the number of traction applications was assessed by calculation of crude odds ratios for two or more and three or more traction applications.

The study protocol was approved by the ethical review board of the hospital. Two-tailed *P* values and confidence intervals were calculated using univariate methods including Fisher's exact test and univariate logistic regression. *P* values of <0.05 were considered statistically significant.

## 3. Results

The characteristics of 87 patients on whom Naegele forceps delivery was performed are presented in Table 1. The proportion of nulliparas in the study group (82.8%) was larger than that in the normal vaginal delivery group (35.1%) in the same period. Maternal complications included gestational diabetes mellitus (11.5%), pregnancy-induced hypertension (10.3%), and psychiatric disorders (4.6%). One case with a previous cesarean history was managed for a trial of labor after cesarean. Fetal head malrotation was diagnosed in 14 (16.1%) cases before forceps delivery, including seven cases each of occiput transverse and occiput posterior position. The numbers of forceps traction applications were one in 64.7% of cases, two in 24.7%, and three or more in 10.7%. The maximum number of traction applications was six. Uterine fundal pressure maneuvers were required only in two (2.3%) cases. The median and maximum traction-to-delivery times were 2 min and 13 min, respectively.

No forceps failure and no slipoff were experienced in the study period, so that the success rate was 100%. No case of shoulder dystocia occurred. Morbidity associated with the forceps deliveries is presented in Table 2. No severe morbidity was seen in the mothers or neonates during short-term observation. Maternal anal sphincter injuries occurred in 35.6%. All cases of anal sphincter injury were appropriately examined and repaired using absorbable sutures without leading to severe problems. Acute postpartum urinary retention lasting over 24 h was seen in 13.8% of cases. All of these cases were eventually resolved by passive catheter bladder drainage. Eight (66.7%) of these cases were resolved within 48 h after delivery. The longest duration to resolution of urinary retention was eight days after delivery. Dehiscence and maternal injuries except for perineal lacerations were seen in five cases (5.7%). They included two cases of pudendal hematoma, two cases of pubic symphysis pain, and one case of wound abscess, which were all resolved in a short period. Neonatal facial injuries were seen in 18.4% of cases. All were mild, and no treatment was indicated. Concerning umbilical artery acidemia, no severe acidemia and no severe neonatal asphyxia were seen. Apgar scores (at 1 and/or 5 min) lower than 7 were not observed.

The relationships between patient characteristic factors and morbidity are described in Table 3. No significant relationship was observed between any of these factors and maternal morbidity. Neonatal facial injury occurred most commonly in cases with malrotation, elevated numbers of traction applications, and maternal complications. Umbilical artery acidemia occurred most commonly in cases with chief indication of nonreassuring fetal status.

**Figure 2:**
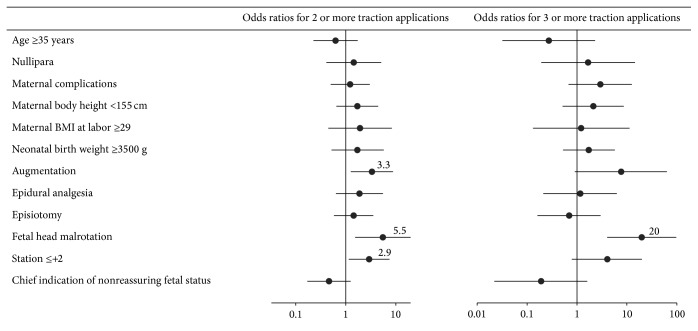
Crude odds ratios for two or more and three or more traction applications. Horizontal bars indicate 95% confidence intervals. BMI: body mass index.

Figure 2 shows the crude odds ratios for two or more and three or more traction applications. Significant odds ratios for two or more traction applications were 5.5, 3.3, and 2.9 in cases with malrotation, augmentation, and station of ≤+2, respectively. Significant odds ratios for three or more traction applications were 20 in cases with malrotation.

## 4. Discussion

All cases were successfully delivered by the Naegele forceps delivery according to the lead author's method as mentioned in the Methods. The forceps delivery was completed with one forceps' traction application in approximately two-thirds of the cases, within two in nearly 90%, and within three in 95%. Malrotation is associated with traction applied three times or more. Malrotation and elevated numbers of forceps traction applications were both related to the occurrence of neonatal facial injury. No severe morbidity was seen in mothers and neonates in the short-term observation.

All the cases were successfully delivered and no forceps failure or slipoff was seen. The success rate of operative vaginal delivery will vary with the chosen type of instrument. In a meta-analysis, O'Mahony et al. [6] reported that forceps delivery was less likely (with risk ratio 0.65) to fail to achieve a vaginal delivery with the chosen instrument than vacuum extraction. In a large-scale retrospective cohort study, Ben-Haroush et al. [9] reported that the failure rates of forceps and vacuum extraction were 1.3% and 10.0%, respectively. Even in cases with failed vacuum extraction, the failure rate of subsequent forceps was 3.5%. Generally, forceps delivery is considered to have a higher success rate than vacuum extraction [6, 10–13]. However, the success rates of operative vaginal delivery will vary with other factors including range of indication, approval for subsequent forceps after failed vacuum extraction, and the operator's proficiency and preference [14].

In this study, forceps delivery was completed with one traction application in approximately two-thirds of cases, within two in nearly 90%, and within three in 95%. In a trial with vacuum extraction, excessive numbers of pulls are considered to increase the risk of neonatal morbidity [10–13]. The guidelines for obstetrical practice in Japan [8] recommend five or fewer pulls in vacuum extraction. In contrast, excessive numbers of forceps traction applications are rarely discussed. There are no recent studies and no recommendations concerning the allowable maximum number of forceps traction applications. Not only in vacuum extraction but also in forceps delivery, multiple traction applications will sometimes be required in cases of dystocia, particularly with fetal head malrotation. In the present study, malrotation seems to be the strongest predictive factor to elevate the number of forceps traction applications. Fetal head malrotation and elevated numbers of forceps traction applications are both risk factors for neonatal facial injury. In the present study, the neonatal facial injuries were mild; therefore, our results may be insufficient to discuss the association between forceps delivery and severe neonatal injuries. However, we believe that our results imply a potential risk for severe neonatal head damage in cases with malrotation and/or elevated numbers of traction applications. Careful attention to the risk factors is thus required.

The rate of maternal anal sphincter injury in forceps deliveries is considered to be approximately 30% [11]. However, the rate will vary with facility and practitioner. Hirsch et al. [15] reported historically two anal sphincter injury rates in their level III teaching hospital. After they promulgated a recommendation to reduce the occurrence of high-degree perineal laceration, the rate of anal sphincter injury with operative vaginal delivery declined from 41% to 26% and that with forceps declined from 40% to 28%. The present lead author usually performs forceps delivery with priority given to delayed disarticulation for smooth fetal head expulsion and midline episiotomy for reducing postsuture pain when episiotomy is needed. There may still be room for measures to reduce the occurrence of anal sphincter injury. Operative vaginal delivery is associated with the occurrence of postpartum urinary retention [16]. Symptoms are brief and are typically resolved within 24 to 48 h of passive catheter bladder drainage [5].

In their large-scale retrospective cohort study, Werner et al. [17] reported that forceps delivery had a lower risk of adverse neonatal outcomes including cephalohematoma, low Apgar score, and neurologic complications and posed a higher risk of facial nerve palsy than did vacuum extraction. In their meta-analysis, O'Mahony et al. [6] reported that facial injury was more likely with forceps but cephalohematoma was more likely with vacuum. Sequential use of vacuum and forceps is associated with increased risk of both maternal and neonatal injury [18]. Relatively low neonatal morbidity and high success rate are the chief reasons for the lead author's choice of forceps for the first-choice instrument.

The management of cases with occiput transverse position is clinically indeterminate. Recently, some researchers have reevaluated the value of Kielland rotational forceps delivery for fetal head malrotation [19, 20]. The lead author uses the method only for cases with deep occiput transverse position, which presents a relatively wide gap within the birth canal to accommodate insertion of the blades of the Kielland forceps and its rotational maneuvering. In cases of dystocia, particularly with malrotation, nonrotational forceps delivery poses a risk of rare and sometimes severe outcomes such as facial nerve palsy [21], depressed skull fracture [22], and corneal abrasion [23]. If the Kielland rotational forceps can be used in the given situation, using it prior to Naegele forceps may reduce the risk of neonatal head damage. The lead author suggests four important points that a forceps operator should recognize: accurate diagnosis of fetal head position including rotation and station, gentle traction, anticipation of potential risks in each case before forceps trial, and decisiveness for halting the forceps trial if descent is not detected.

As described here, the lead author gives priority to forceps delivery for operative vaginal delivery. In the author's opinion, particularly for the fetus and neonates, excessive stress can be avoided not only in cases with successful forceps delivery but also in cases with failed forceps delivery, as compared to cases with vacuum extraction. The reasons are relatively short traction-to-delivery time, no need of uterine fundal pressure maneuvers, and even in failure cases the possibility of an early decision to halt the forceps trial. Excessive pulls of vacuum extraction with uterine fundal pressure maneuvers may lead to infant cerebral palsy and uterine rupture [24]. We hope that the value of forceps delivery will be rerecognized and that many obstetric residents will be given the chance of training in the method and technique of forceps delivery.

## Figures and Tables

**Table 1:** Characteristics of the 87 pregnant women on whom Naegele forceps delivery was performed.

Factors	Median	Range	*n*	%
Gestational age at delivery (weeks)	39 5/7	37 3/7–41 4/7		
Early term (37 0/7-38 6/7 weeks)			19	21.8
Full term (39 0/7-40 6/7 weeks)			56	64.4
Late term (41 0/7 weeks and after)			12	13.8
Age (years)	32	19–40		
Parity				
Nullipara			72	82.8
Para 1			11	12.6
Para 2			4	4.6
Maternal complications			37	42.5
Maternal height (cm)	158	147–170		
Maternal weight at labor (kg)	59.6	44.7–91.8		
Maternal BMI at labor (kg/m^2^)	24.2	18.6–34.8		
Neonatal birth weight (g)	3036	2072–3926		
Augmentation			48	55.2
Epidural analgesia			18	20.7
Episiotomy			35	40.2
Midline			24	27.6
Mediolateral			11	12.6
Fetal head malrotation			14	16.1
Chief indication				
Prolonged second stage			51	58.6
Nonreassuring fetal status			33	37.9
Severe PIH			3	3.4
Station (cm)				
+1			2	2.4
+2			41	47.7
+3			30	34.9
≥+4			13	15.1
Missing			1	
Numbers of forceps traction applications				
1			55	64.7
2			21	24.7
3			5	5.9
4			2	2.4
5			1	1.2
6			1	1.2
Missing			2	
Uterine fundal pressure maneuver			2	2.3
Traction-to-delivery intervals (min)	2	0–13		

BMI: body mass index; PIH: pregnancy-induced hypertension.

**Table 2:** Morbidity associated with Naegele forceps deliveries.

Morbidity	*n*	%
Maternal morbidity		
Postpartum hemorrhage >500 mL	5	5.7
Blood transfusion	1	1.1
Perineal laceration		
None	0	0.0
1st degree	2	2.3
2nd degree	54	62.1
3rd degree	26	29.9
4th degree	5	5.7
Acute postpartum urinary retention lasting over 24 h	12	13.8
Dehiscence and maternal injury except for perineal lacerations	5	5.7
Neonatal morbidity		
Facial injuries	16	18.4
Cephalohematoma	3	3.4
Umbilical artery acidemia		
Umbilical artery pH: 7.10–7.19	8	9.2
Umbilical artery pH: <7.1	0	0.0
Neonatal intensive care unit admission	1	1.1

**Table 3:** Relationship between patient characteristics and morbidity.

Factors	Maternal anal sphincter injury (*n* = 31)		Acute postpartum urinary retention lasting over 24 h (*n* = 12)		Dehiscence and maternal injury except for perineal lacerations (*n* = 5)		Neonatal facial injuries (*n* = 16)		Umbilical artery acidemia (*n* = 8)	
	%	*P*	%	*P*	%	*P*	%	*P*	%	*P*
Gestational age at delivery										
Early term (37 0/7-38 6/7 weeks)	35.7	0.85	16.7	0.73	5.3	>0.99	25.0	0.56	10.5	>0.99
Full term (39 0/7-40 6/7 weeks)	31.6		12.5		7.1		26.3		8.9	
Late term (41 0/7 weeks and after)	41.7		16.7		0.0		16.7		8.3	
Maternal age										
≥35 years	48.0	0.15	11.3	0.31	4.0	>0.99	12.0	0.54	4.0	0.43
<35 years	30.7		20.8		6.5		21.0		11.3	
Parity										
Nullipara	37.5	0.56	12.7	0.44	6.9	0.39	20.8	0.29	9.3	>0.99
Multipara	26.7		20.0		0.0		6.7		6.7	
Maternal complications										
Yes	27.0	0.15	16.7	0.55	2.7	0.30	32.4	0.005^*∗*^	5.4	0.46
No	42.0		12.0		8.0		8.0		12.0	
Maternal height										
<155 cm	36.0	>0.99	12.0	>0.99	8.0	0.63	28.0	0.22	4.0	0.43
≥155 cm	35.5		14.8		4.8		14.5		11.3	
Maternal BMI at labor										
≥29 kg/m^2^	50.0	0.46	37.5	0.064	0.0	>0.99	12.5	1	0.0	>0.99
<29 kg/m^2^	34.6		10.4		6.4		19.2		10.3	
Neonatal birth weight										
≥3500 g	35.7	>0.99	7.1	0.70	0.0	0.59	21.4	0.72	7.1	>0.99
<3500 g	35.6		15.3		6.9		17.8		9.6	
Augmentation										
Yes	43.8	0.12	10.3	0.54	6.3	>0.99	25.0	0.099	6.3	0.46
No	25.6		17.0		5.1		10.3		12.8	
Epidural analgesia										
Yes	38.9	0.79	22.2	0.27	1.1	0.28	22.2	0.74	5.6	>0.99
No	34.8		11.8		4.4		17.4		10.1	
Episiotomy										
Yes	40.0	0.51	5.7	0.12	8.6	0.39	20.0	0.79	2.9	0.14
No	32.7		19.6		3.9		17.3		13.5	
Fetal head malrotation										
Yes	42.9	0.56	7.1	0.69	0.0	0.59	64.3	<0.001^*∗*^	7.1	>0.99
No	34.3		15.3		6.9		9.6		9.6	
Station										
≤+2 cm	27.9	0.26	14.3	>0.99	4.7	>0.99	20.9	0.79	11.6	0.71
>+2 cm	41.9		14.0		7.0		16.3		7.0	
Chief indication										
Prolonged second stage	43.1	0.11	16.0	0.85	5.9	>0.99	23.5	0.053	2.0	0.0053^*∗*^
Nonreassuring fetal status	24.2		12.1		3.0		6.1		26.9	
Severe PIH^†^	33.3		0.0		0.0		66.7		0.0	
Numbers of forceps traction applications										
1	34.5	0.58	16.7	0.58	7.3	>0.99	7.3	<0.001^*∗*^	5.5	0.14
2	42.9		14.3		4.8		19.0		14.3	
≥3	22.2		0.0		0.0		88.9		22.2	

Percentages mean proportions of the morbidity in each factor. *P* values were calculated using Fisher's exact test.

BMI: body mass index; PIH: pregnancy-induced hypertension.

^*∗*^Statistically significant.

^†^Chief indication of severe PIH was excluded from the statistical test because of the lack of the cases (*n* = 3).
